# 3D isotope density measurements by energy-resolved neutron imaging

**DOI:** 10.1038/s41598-022-10085-3

**Published:** 2022-04-22

**Authors:** A. S. Losko, S. C. Vogel

**Affiliations:** 1 Los Alamos National Laboratory, Los Alamos, NM 87545 USA; 2 Forschungs-Neutronenquelle Heinz Maier-Leibnitz, 85748 Garching, Germany

**Keywords:** Experimental nuclear physics, Characterization and analytical techniques, Nuclear fuel

## Abstract

Tools for three-dimensional elemental characterization are available on length scales ranging from individual atoms, using electrons as a probe, to micrometers with X-rays. However, for larger volumes up to millimeters or centimeters, quantitative measurements of elemental or isotope densities were hitherto only possible on the surface. Here, a novel quantitative elemental characterization method based on energy-resolved neutron imaging, utilizing the known neutron absorption cross sections with their ‘finger-print’ absorption resonance signatures, is demonstrated. Enabled by a pixilated time-of-flight neutron transmission detector installed at an intense short-pulsed spallation neutron source, for this demonstration 3.25 million state-of-the-art nuclear physics neutron transmission analyses were conducted to derive isotopic densities for five isotopes in 3D in a volume of 0.25 cm^3^. The tomographic reconstruction of the isotope densities provides elemental maps similar to X-ray microprobe maps for any cross section in the probed volume. The bulk isotopic density of a U-20Pu-10Zr-3Np-2Am nuclear transmutation fuel sample was measured, agrees well with mass-spectrometry and is evidence of the accuracy of the method.

## Introduction

Characterizing the elemental composition of materials is of great interest in scientific disciplines ranging from mineralogy to energy materials. Well established methods like X-ray fluorescence^1^ provide *elemental* compositions on the surface and methods such as laser ablation inductively coupled plasma mass spectrometry^2^ allow *isotopic* concentration measurements on the surface. Knowledge of the *three-dimensional* elemental concentration is desirable in many cases and electron- and X-ray based methods were developed for nanometer^3^ and micrometer^4,5^ length scales. Destructive methods, analyzing nanometer scale volumes by removing atom by atom, also exist^6^. All of these techniques require significant sample preparation. While millimeter to centimeter scale 3D visualizations of cracks and average attenuation, without elemental sensitivity, is provided by hard X-ray^7,8^ or thermal neutron tomography^9^, a method for three-dimensional element or isotope density measurements on millimeter or centimeter length-scales was hitherto missing.

Here, a novel quantitative method is presented based on energy-resolved neutron imaging, utilizing the known neutron absorption cross sections with their ‘finger-print’ absorption resonance signatures. First measurements by Sato et al. showed that neutron absorption resonance spectroscopy with computer tomography can non-destructively provide nuclide density inside an object by analyzing transmitted intensities at absorption resonance energies for specific isotopes^10^. In this first approach, applicability of the method was limited due to only few projections recorded with low spatial resolution, i.e. 2.3 mm provided by a 64 (8 × 8) pixel ^6^Li-glass scintillation counter. However, the authors alluded to the potential for quantitative elemental analysis. Utilizing more advanced detector technology, state-of-the-art hardware and software solutions we report, based on 3.25 million state-of-the-art nuclear physics neutron transmission analyses, isotopic densities for five isotopes in 3D in a volume of 0.25 cm^3^. The method presented in this work is enabled by a pixilated time-of-flight (ToF) neutron transmission detector with 512 × 512 pixels and a pixel size of 55 × 55 μm^2^^11^ installed at an intense short-pulsed spallation neutron source^12^. Like similar non-isotope specific neutron techniques, the method does not require sample preparation and works with hazardous samples enclosed in containers.

Energy-resolved neutron imaging is often applied to the cold or thermal neutron energy range to gain crystallographic or phase information via Bragg-edge imaging methods^13–16^. In this energy range, the attenuation of neutrons by most isotopes is dominated by elastic scattering, limiting the capability for extracting densities reliably, particularly if the material contains multiple elements or the crystals have a preferred orientation (texture). In contrast, utilizing neutron absorption resonances as unique ‘finger-prints’ of isotopes in the epithermal neutron energy range allows to reliably determine the 2D areal-density distribution of isotopes with suitable resonances that can be resolved with the energy resolution provided at the instrument. This is independent of the atomic arrangement, i.e. crystal structure, of the atoms and of the microstructure of the material, i.e. texture, phase fractions etc. Well-developed neutron cross section analysis implemented in state-of-the-art neutron transmission data analysis codes^17,18^ allows to determine the areal densities of multiple isotopes simultaneously, including considerations such as self-attenuation or resonance interference described by the Reich–Moore formalism^19^. At a short-pulse neutron source, such as the Los Alamos Neutron Science Center (LANSCE)^12^, pixilated time-of-flight neutron detectors^11^ enable therefore three-dimensional isotope density measurements as the number of nuclei of a given isotope can be measured per voxel using tomographic data-sets. Previous research has also estimated the isotope density in two dimensional radiographs^20,21^, and alluded to the potential of this technique^22,23^. However, the massive amount of neutron transmission analyses for each pixel recorded in a tomography dataset consisting of several tens or hundreds of rotations, strategies for proper background treatment to obtain reliable results and other obstacles have prevented full development of this intriguing technique. With this work, we demonstrate that three-dimensional isotope density measurements are possible with energy-resolved neutron imaging.

## Energy-resolved neutron imaging of nuclear fuel slugs

Using energy-resolved neutron imaging, a U-20Pu-10Zr-3Np-2Am (weight percent) sample, an alloy researched for transmutation nuclear fuels^24^, was characterized to obtain the bulk composition for comparison with mass-spectrometry. The sample consisted of a 20 mm long, 4.2 mm diameter U-20Pu-10Zr-3Np-2Am (weight percent) cast fuel slug contained in a double-walled steel container. The sample was prepared at Idaho National Laboratory where also mass spectrometry was performed (Table 1). The sample was mounted on a rotation stage and energy-resolved neutron imaging data was collected for 120 min per rotation for 65 rotations.

**Table 1:** Isotope densities from energy-resolved neutron imaging compared with those from mass spectrometry. For the neutron analysis, the fractional density was computed by the average of the reconstructed sample volume, averaged over all voxels fully inside the sample with errors computed using the standard deviation of the voxel densities.

Isotope	Weight fraction from mass spectrometry (μg/g)	Fractional density from mass spectrometry (g/cm^3^)	Fractional density from neutron analysis (g/cm^3^)
^234^U	< 30	0.00	
^235^U	1490	0.02	
^236^U	113	0.00	
^237^Np	24,400	0.34	0.310 (3)
^238^U	639,000	9.01	10.8 (2)
^239^Pu	166,000	2.34	2.26 (2)
^240^Pu	26,400	0.37	0.369 (3)
^241^Am	23,000	0.32	0.314 (3)
Zr	100,000	1.41	
Total	980,433	13.82	14.09

**Figure 1:**
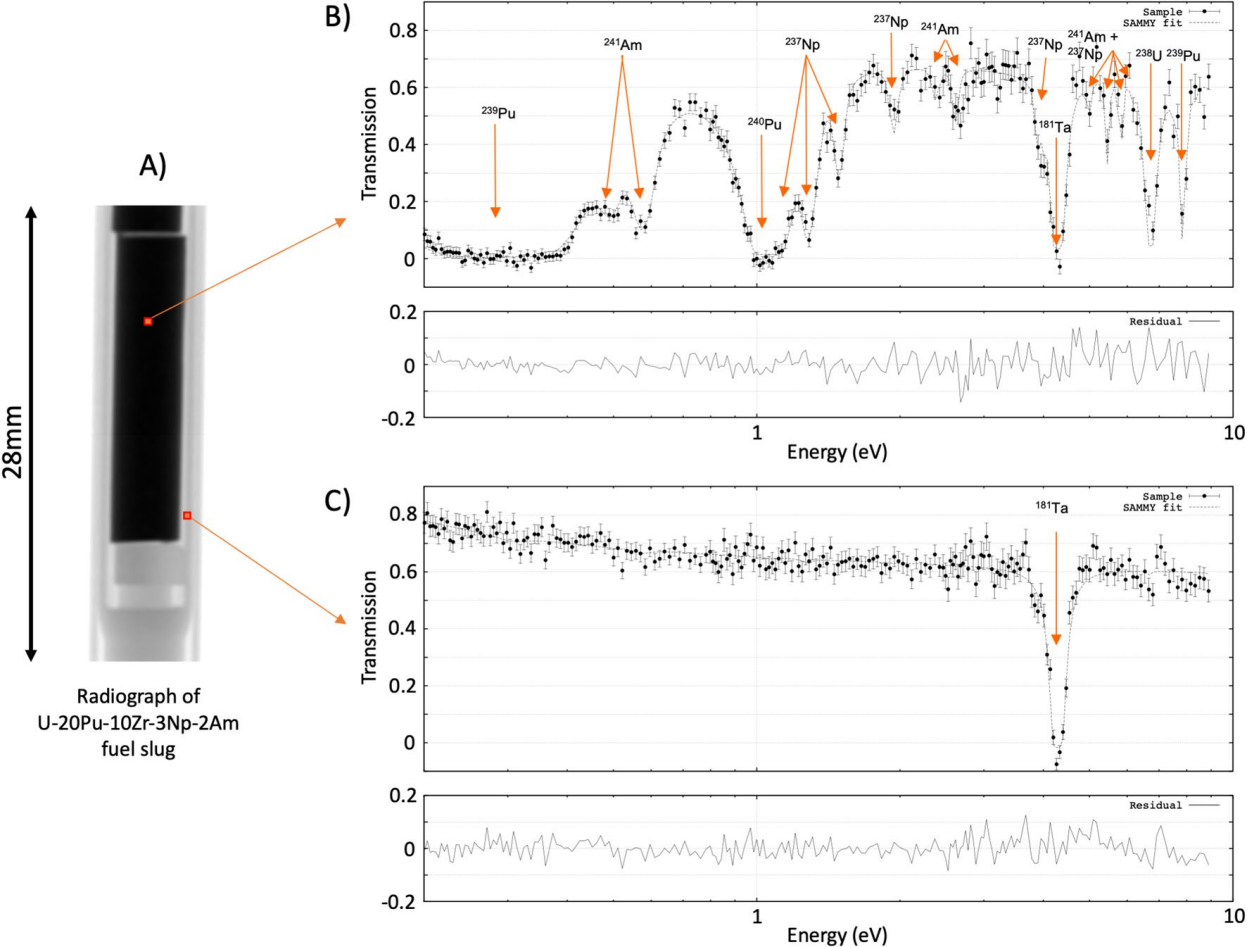
Thermal neutron radiograph (**A**) with single pixel data for isotope concentration measurement with a fit of the transmission data inside (**B**) and outside (**C**) the sample. Arrows mark the resonances of several isotopes and the difference curves between experimental data and fit are shown below.

Considering only an active-area on the detector from pixels where the neutron beam traversed the sample volume (Fig. 1A), 50,000 (125 × 400) pixels required neutron transmission data analysis per sample rotation, resulting in 3.25 million total transmission-spectra fits. The SAMMY code^17^ was used with cross section data for the isotopes ^237^Np, ^238^U, ^239^Pu, ^240^Pu, and ^241^Am obtained from the ENDF/B-VIII.0 data base^25^. For conventional neutron transmission measurements, the sample is generally several tens of meters away from the detector, such that sample induced background can be neglected^26^. In contrast, for imaging measurements, the sample position is as close as possible to the detector to reduce blurring resulting from the divergent beam. However, this sample position impacts the background in imaging applications and therefore the sample induced background needed to be properly accounted for to obtain reliable areal and volumetric densities. To accomplish this, a Ta foil was mounted on the detector window with a thickness of 100 μm, leading to opaque resonances. This allowed for reliable determination of the background, including sample contributions, by the transmission values at the bottom of these resonance dips in the transmission data.

Figure 1 shows an example of the data analysis for single pixels with fitted transmission for a pixel within the slug in (B) and outside the slug in (C). The total computing time for the data analysis of the tomographic data-set processing 3.25 million spectra on a desktop PC using 64 × 2.4 GHz CPUs was 16 days. The bulk composition was determined from the average density of the reconstructed sample volume. Table 1 shows the comparison of the results with the results of the mass spectrometry. The maximum discrepancy for the four isotopes except ^238^U is 0.08 g/cm^3^, establishing the reliability of this method. The reason for the discrepancy of 1.8 g/cm^3^ for ^238^U lays in the properties of the ^238^U resonance in the dataset. The analyzed energy range of 0.2–9 eV only provided a single ^238^U absorption resonance at 6.67 eV. At a fractional density of ~ 9 g/cm^3^ and cross sections ≫ 10^3^ barn, this led to insufficient non-zero transmission at resonance energies for a reliable data fit. Furthermore, the absorption resonance at 6.67 eV for ^238^U has a comparatively broad profile with respect to the other isotopes present and the resonance overlaps significantly in the non-zero transmission range with other resonances. Increasing the energy range to include more ^238^U resonances for the analysis could mitigate this problem but was not feasible with the available resources for all 3.25 million datasets.

Additionally, It should be noted that despite some absorption reasonances providing broader profiles than others, e.g. ^239^Pu provided the broadest and most opaque resonance, accuracy in isotope concentration or quality of reconstruction was not heavily impacted by the resonance widths of the different isotopes present in this sample. This can be explained by the proportionality between integrated area for the attenuation by a specific resonance and the number of atoms for the specific isotope in the beam path remaining intact despite broadening. Therefore, as long as the absorption resonances can be measured with a sufficiently large number of time-bins for proper profile fitting and are not severely overlapped by resonances from other isotopes, the impact of energy resolution on the accuracy of the isotope quantification is negligible. These two conditions are of course affected by the time resolution of the detector system (several µs in our case^27^) as well as by the energy resolution of the pulsed source utilized (e.g. the initial proton pulse width producing spallation neutrons should be negligible relative to the moderation time).

**Figure 2:**
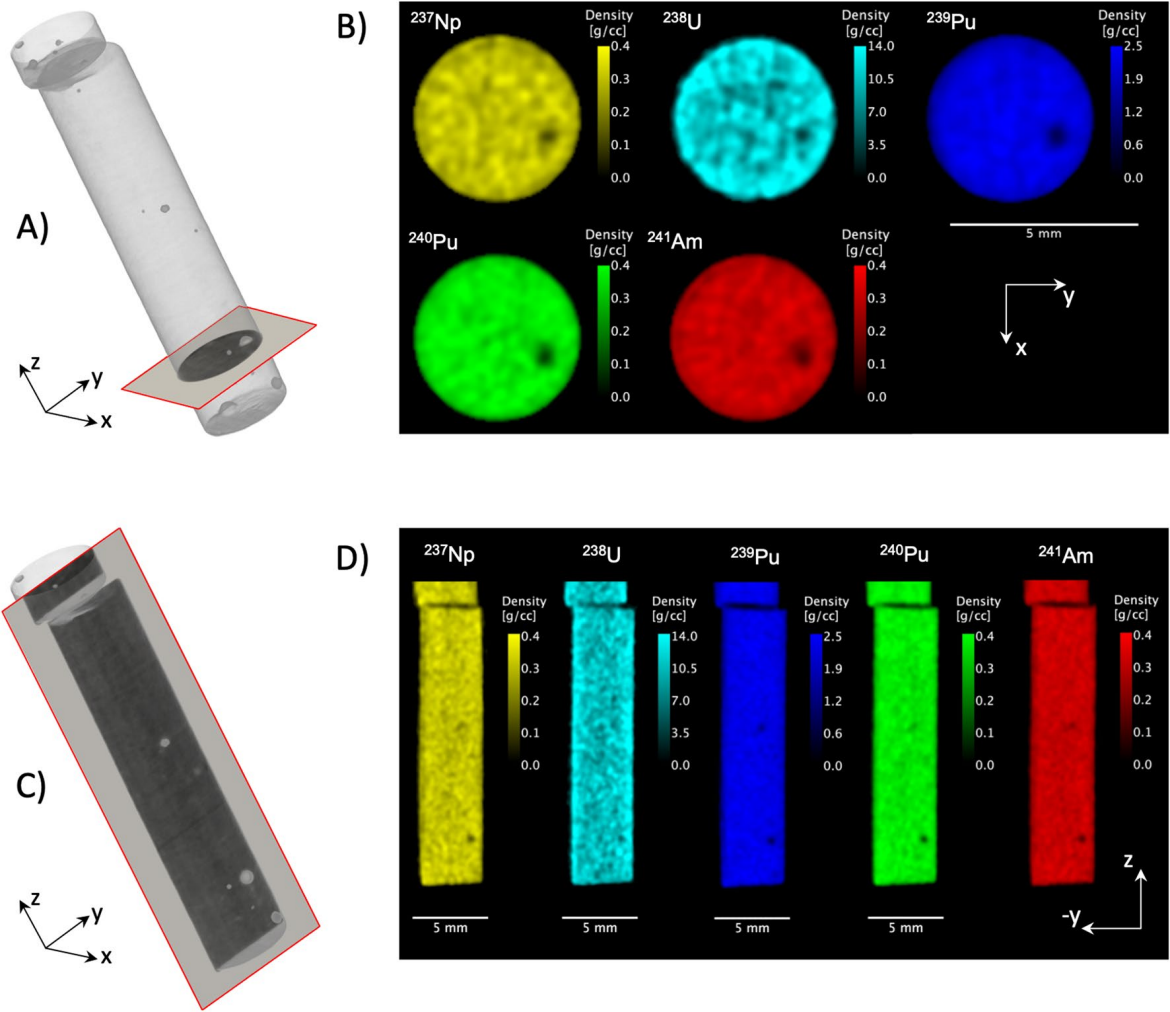
Volumetric reconstruction using epithermal neutrons of the U-20Pu-10Zr-3Np-2Am sample with indicated region in red for CT slices of the volumetric densities of individual isotopes. Slices normal to the cylinder axis in (**A**) with corresponding isotope densities shown in (**B**) and slices parallel to the cylinder axis in (**C**) with corresponding isotope densities shown in (**D**), respectively.

The large absorption cross section for thermal neutrons of ^239^Pu made it impossible to utilize thermal neutrons for neutron tomography. Using the sample composition, the computed transmission at a thickness of 4.2 mm for 25 meV neutrons equates to 3.5%. For such opaque samples, the reconstruction leads to artefacts of the average voxel density, so-called ‘beam hardening’^28^. However, at epithermal neutron energies (*E* > 0.4 eV) the cross section drops such that significant fractions of the beam are transmitted and a reliable tomographic reconstruction is possible. Figure 2 shows a 3D rendering of the fuel slug resulting from neutron tomography by selecting time-of-flight neutrons only at the upper end of the thermal spectrum, with energies ranging from 0.1 to 0.2 eV. Several globular features are apparent. The tomographic reconstruction of the densities of the aforementioned isotopes allowed further investigation of these features and greatly reduced densities for all isotopes were found at the locations of the globular features. This would indicate that the features are either casting voids or Zr-rich metallic inclusions (Zr did not provide neutron absorption resonances in the 0.2–9 eV energy range used for the measurements). With the slices of the tomographic reconstruction providing data similar to elemental maps provided by X-ray microprobe, but within the bulk of a sample, non-destructively, and for samples that have to be in containers such as nuclear fuels, this characterization guides destructive examination by identifying where this sample should be cut for further investigations.

## Summary and conclusions

In summary, three-dimensional quantitative isotope density measurements on cm^3^ sized volumes with sub-mm spatial resolution were demonstrated with the results in agreement to prior measurements using mass spectrometry. This technique extends the range of available elemental or isotope-density measurement techniques to the mm length scale. At present, applications include characterization of fresh and irradiated nuclear fuels (as demonstrated here), dopant concentrations of large crystals for scintillator applications^21^, fission gas pressure measurements^29^ and measurements of ﻿isotope-specific ion uptake from aqueous solutions into concrete^30^. All of these rely on the sample containing elements with suitable neutron absorption resonances for this technique. A combination of a short-pulsed neutron source with a pixilated time-of-flight neutron imaging detector and massive data analysis runs with state-of-the-art nuclear physics analysis code enabled this unique capability.

Artefacts, such as beam hardening, for samples that attenuate the beam significantly over a large energy range, can be eliminated by the presented technique, requiring only a subset of neutron energies that provide significant non-zero transmission for the SAMMY code^17^ to determine accurate areal densities. This has been illustrated in this work in the case of ^239^Pu, providing accurate reconstruction despite significant attenuation of the beam by the ^239^Pu absorption resonances.

Accurate determination of isotopic densities with the presented approach relies on multiple aspects, such as number and cross-section of absorption resonances for a given isotope, overlap of absorption resonances of different isotopes and last but not least the flux of neutrons at the specific energies. Additionally, while not only the flux for epithermal neutrons using a moderated source decreases with increasing neutron energy, the detection efficiency of the deployed detector also significantly reduces based on the ^10^B capture efficiency for epithermal neutrons^27^. This limits this technique at present to absorption resonances below 100 eV for tomographic measurements due to the significant increase in acquisition time for absorption resonances at higher energies.

A recent development in scintillator-based neutron detection^31^ offers new perspectives for the presented technique and could overcome some of the limitations with the detector deployed in this work. This new development provides a neutron counting principle based on a detector that offers a variable field of view (roughly 1 × 1 cm^2^ to 20 × 20 cm^2^) and interchangeable scintillators. For example, utilizing thick scintillators would allow to significantly increase detection efficiency compared to the ^10^B doped sensor^11,27^, particularly benefiting the epithermal neutron regime. Furthermore, a major challenge in the presented work was the determination of the background, which is predominantly caused by gamma contributions. Neutron/gamma discrimination enabled by this novel technique would therefore allow to suppress such background^31^. Extending the accessible energy range with an improved background would significantly enlarge the potential applications of the presented technique by allowing to access a larger number of isotopes with resonances at higher neutron energies than presently accessible. Software improvements, e.g. to identify isotopes present in the spectra based on a library of cross-sections and further improving background subtraction are on-going.

The method described here offers potential in any field where at present techniques, such as inductively coupled plasma mass spectrometry (ICP-MS), are applied for isotope analysis with isotopes that exhibit neutron absorption resonances and samples of at least a few millimeters in size. The technique described here would increase the probed volume by orders of magnitude as well as provide a spatial distribution of the isotopes. The neutron-based technique complements X-ray or electron-based micrometer resolution techniques for elemental identification, allowing e.g. to identify regions of interest in cubic centimeters of irradiated nuclear fuel during advanced fuel development. While ordering processes in alloys occur on tens of nanometer length scales, e.g. leading to characteristic lamellar microstructures, will not be accessible with this technique, larger length scale processes such as during fractional solidification in alloy casting, geoscience applications or molten salts like NaCl-UCl_3_ mixtures could be characterized with the technique described here. For nuclear applications, including nuclear forensics, the ability to distinguish between fissile ^235^U and ^238^U goes beyond what X-ray and electron-based methods offer and has the advantage over mass-spectrometry that the characterization can be accomplished with the sample sealed inside shielding containers.

Finally, while limited at the present time to large-scale pulsed neutron user facilities, the potential of laser-driven short pulse intense neutron sources^32,33^ may provide this technique at other facilities within a decade.

## Methods

At the pulsed spallation neutron source at LANSCE, 270 ns long (base-to-base) pulses of 800 MeV protons from a linear accelerator and proton storage ring produce neutron bursts at 20 Hz which are subsequently moderated. Collimation consisted of steel and borated polyethylene rings increasing from 2 cm inner diameter to 5 cm over a total length of 150 cm with a distance of ~ 750 cm from the smallest diameter of the collimator to the sample (see^34^ for more details on the beam line). The neutrons in each pulse arrive at the detector sorted by their energy, with higher energy neutrons arriving first. The time-of-flight from the moderator to the detector allows with the calibrated distance between moderator and detector to compute the velocity and kinetic energy of the neutron^35^. Tabulated neutron interaction cross sections are available in databases such as ENDF^36^. Many isotopes, especially isotopes heavier than ~ Zr, exhibit sharp increases in their neutron absorption cross section for specific neutron energies, so-called neutron absorption resonances^37–39^. Similar to optical spectroscopy, where an element can be identified by its optical emission or absorption spectrum, neutron resonance spectroscopy allows to identify isotopes present in transmission spectra by means of neutron resonance transmission analysis^40^. The neutron transmission *T* for neutrons of energy *E* is given by

1 $$T\left(E\right)=\frac{{I}_{s}\left(E\right)-{I}_{bs}(E)}{{I}_{o}\left(E\right)-{I}_{bo }(E)}={exp}^{-\sum_{i}{n}_{i}{\sigma }_{i}(E)},$$

whereby *I*_*s*_*(E)* is the intensity transmitted through the sample, *I*_*bs*_*(E)* the background with sample in beam, *I*_*o*_*(E)* the incident or open beam intensity and *I*_*bo*_*(E)* the background for the open beam. All of these intensities vary for each pixel of a 2D detector and therefore need to be measured pixel by pixel. In the exponent, the sum is running over each isotope present in the sample, with *n*_*i*_ the areal density of the *i*_*th*_ isotope, and *σ*_*i*_*(E)* the cross section of the *i*_*th*_ isotope for neutrons of energy *E*^26,41^. For the purpose of isotope density measurements, the cross section is assumed to be known, leaving the areal density as the only variable during the analysis using the SAMMY code^17^. This analysis has to be performed for each pixel of the detector where the sample is visible. In our case, a detector consisting of a multi-channel plate and a MediPix readout chip was utilized^11^ to record ~ 3000 frames per 50 ms pulse of the neutron source with a pixel size of 55 μm. The field of view is 512 × 512 pixels or 28.16 × 28.16 mm^2^.

Errors in the transmission spectra for each pixel as shown in Fig. 1B,C were determined based on Poisson counting statistics for each pixel, i.e. sqrt(*I*) where *I* is the intensity. Each particle that is detected by the sensor activates on average ~ 4 pixels, incrementing the activated pixels by 1 grey-value. Individual frames corresponding to different time-of-flight were integrated for the duration of the experiment. For each frame, event overlap (pixel dead time) was negligible. Therefore, as an approximation, the total number of particles measured for each frame and pixel was assumed ~ 1/4 of the measured grey-value. Finally, applying error propagation to Eq. (1), absolute errors for the transmission values *T(E)* were determined.
